# Healthcare providers’ perceived barriers to providing breastfeeding support in Northwest rural China

**DOI:** 10.1186/s13006-024-00630-3

**Published:** 2024-04-03

**Authors:** Jiao-jiao Wu, Qing-ning Zhang, Su-su Liao, Jiang-hong Li, Jian-duan Zhang, Jing-zhi Huang

**Affiliations:** 1 School of Nursing, Lanzhou University, Yanxi Road 28, Lanzhou, Gansu China; 2 School of Philosophy and Sociology, Lanzhou University, 222 Tianshuinan Road, Lanzhou, Gansu China; 3 Institute of Basic Medical Science, Chinese Academy of Medical Sciences & Peking Union Medical College, No. 5 Dongdan Santiao, Beijing, China; 4 Institute for Community Research, 2 Hartford Sq. W., Ste. 210, 06106 Hartford, CT USA; 5 Department of Maternal and Child Health, School of Public Health, Tongji Medical College, Huazhong University of Science and Technology, 13 Hangkong Rd, Wuhan, Hubei China; 6 Graduate School of Interdisciplinary Information Studies, The University of Tokyo, 7-3-1 Hongo, Bunkyo, Tokyo Japan

**Keywords:** Breastfeeding, Healthcare provider, Perceived barrier, Rural China, Qualitative research

## Abstract

**Background:**

Healthcare providers play important roles in supporting breastfeeding. Although there has been insufficient actual breastfeeding support from healthcare providers in China, little research has been conducted to understand Chinese healthcare providers’ perceived barriers to providing breastfeeding support, especially in rural China. This study aims to identify these perceived barriers to providing breastfeeding support in Northwestern rural China.

**Methods:**

This study was conducted during the period from March 2018 to December 2018. Forty-one healthcare providers were recruited through purposive sampling in two rural counties in Northwest China that are in close proximity to each other and share similar demographic features. Participants included obstetrician-gynecologists, midwives, nurses, “village doctors”, and township and village maternal and child health workers. Qualitative data were collected through one-on-one in-depth semi-structured interviews and focus group discussions. Transcripts were thematically analyzed.

**Results:**

Analysis of interview data resulted in four themes that the participants perceived as barriers to supporting breastfeeding: (1) lack of medical resources, within which inadequate staffing, and lack of financial incentives were discussed, (2) lack of clear and specific responsibility assignment, within which no one takes the lead, and mutual buck-passing were discussed, (3) healthcare providers’ lack of relevant expertise, within which lack of knowledge and skills, and low prestige of village healthcare providers were discussed, (4) difficulties in accessing mothers, within which medical equipment shortages reduce services utilization, mothers’ housing situation, mothers’ mobility, and cultural barriers were discussed.

**Conclusions:**

The study identified HCPs perceived barriers to providing breastfeeding support. Unique to China’s Tri-Level Healthcare System, challenges like staffing and financial incentives are hard to swiftly tackle. Recommendations include mHealth enhancement and clarified responsibilities with incentives and tailored training. Further research is crucial to evaluate these strategies in rural Northwestern China and comparable underdeveloped areas nationwide.

## Background

The health benefits of breastfeeding for both mother and baby are widely acknowledged by academics and healthcare providers. The Chinese government introduced the Baby Friendly Hospital Initiative in the 1990s. Despite the efforts by government and organizations such as UNICEF in promoting breastfeeding knowledge and practices, China’s breastfeeding rates are still far from satisfactory [1]. The Chinese Nutrition and Health Surveillance in 2013 revealed that the national rates of exclusive breastfeeding were 20.8% at 6 months, 11.5% at 1 year, and 6.9% at the 2-year mark [2]. These results are half of the global 6-month average and fall well short of the goals of China’s National Programme of Action for Child Development 2021–2030, which aims to achieve a breastfeeding rate of more than 50% for infants between 0 and 6 months [3]. The western regions of China are at an even greater disadvantage in terms of maternal health services, particularly in rural areas [4]. Studies have shown that in rural western regions, the exclusive breastfeeding rates for Han, Uygur, Tibetan, and Zhuang groups at 6 months are 11.6%, 0.8%, 4.4%, and 13.8%, respectively [5, 6].

Various healthcare providers (HCPs) such as midwives, nurses, obstetrician-gynecologists, paediatricians, and lactation consultants have played important roles in supporting breastfeeding worldwide [7, 8]. The specific breastfeeding support responsibilities of HCPs are well defined in countries like the United States and Australia. In China, HCPs collectively are also required to provide breastfeeding support to mothers and infants across the prenatal to postpartum continuum. However, the specific supporting roles and responsibilities of the various providers are not clearly defined [9]. Further, HCPs in China have not provided sufficient actual breastfeeding support on the ground [10, 11].

HCP’s experiences and perceived barriers to supporting breastfeeding are critical factors that policymakers and managers should consider when developing measures to promote breastfeeding. A variety of barriers affecting HCP’s breastfeeding support have been identified in other countries, including cultural barriers, policies and practices unsupported by healthcare institutions, lack of coordination and continuity across HCPs, inadequate staffing, HCP time constraints, HCP resistance to taking responsibility for breastfeeding support, insufficient HCP breastfeeding support expertise, poor infant health, and mothers’ poor health (including mental health) [12–18]. Further, research on breastfeeding support that is conducted on Chinese HCPs is mainly quantitative, and focuses on their breastfeeding knowledge and breastfeeding interventions in urban areas [19, 20]. Little research has been conducted to understand HCP perceived barriers to the provision of breastfeeding support, especially the case in the rural Chinese healthcare system.

This paper focuses on data collected through the qualitative component of a larger study that was sponsored by UNICEF in 2018 entitled “The anthropological study on breastfeeding in rural areas of Northwest China”. The main research goal in this component of the study was to gain more in-depth understanding of HCP perspectives on barriers encountered in the provision of breastfeeding support across the prenatal to postpartum continuum, within the complex context of China’s rural healthcare system.

## Methods

The original study included surveys, in-depth interviews (IDIs), and focus group discussions (FGDs) with HCPs, mothers, and grandmothers. This paper utilizes the IDIs and FGDs data to explore HCP’s perspectives.

### Setting

The study was conducted in Datong County, Qinghai Province and Yanchi County, Ningxia Province. These two counties are both impoverished with limited health care facilities in Northwest China at the time of the study. Datong County is located in the transition zone between the Qinghai Tibetan and the Chinese Loess Plateaus, at an altitude of 2,280-4,622 m, covering a total area of 3,090 square km. The entire county is dominated by mountains, being surrounded by mountains on three sides. Datong County has a total population of 403,400 people comprising 31 ethnical groups, namely Han (mainstream Chinese) and ethnic minority groups such as the Hui, Tu, and Tibetans. Yanchi County covers a total area of 8,522.2 square kilometres, with hills as the main landform. The total population of Yanchi county is 173,000, including an agricultural population of 143,000 and more than 4,000 members of ethnic minorities (primarily Hui).

Similar to that of thousands of counties in China, healthcare services in Datong and Yanchi, are provided by the Tri-Level Healthcare System, which oversees county-level hospitals, county Maternal Child Health (MCH) centres, township health centres, and village clinics. County-level hospitals, the tertiary level of the system, provide general medical care to the county population, including women and children. They also accept referrals from township health centres and village clinics, and manage medical emergencies and critical illnesses. There are three county-level hospitals in Datong; two of them are certified as Baby-Friendly Hospitals. In Yanchi, there are two county-level hospitals, with one of them certified as a Baby-Friendly Hospital. In both counties, almost all babies are born at county-level hospitals, with three days of hospital stay after vaginal birth and seven days after a cesarean section, unless complications require a longer stay. The Baby-Friendly Hospitals are required to provide breastfeeding services following the guidelines defined in the Ten Steps to Successful Breastfeeding and the WHO Code for the Marketing of Breast Milk Substitutes, which aim to support, protect, and promote breastfeeding. There are county MCH centres in both Datong and Yanchi, which are mainly responsible for public health services such as health education, coordination and health data monitoring for women and children in the county. With regard to breastfeeding, the county MCH centres mainly collect breastfeeding rates and supervise breastfeeding support provided by other medical institutions.

Township health centres, the secondary level of the health system, provide clinical care for common diseases and basic public health services to residents in the township jurisdictional area. All pregnant women are required to register at the township health centre within 12 weeks of pregnancy. Funded by the Chinese government, township health centres are required to provide pregnant women with five prenatal checkups and one postpartum checkup (42 days after giving birth) free of charge. There is a full-time but inadequately trained MCH worker in each county’s township health centre, who is mainly responsible for the public health services of women and children in the township.

Village clinics, the primary level of the system, provide basic healthcare and public health services to the village residents. The village HCPs in both counties are part-time village residents. They are inadequately trained in medicine, certified by local health authorities with the so-called title of “village doctor” or “MCH worker”, rather than a regular HCP license. In each Datong village, there is one village doctor and one female MCH worker. The MCH workers are required to conduct the first postpartum visit within seven days and the second on the 28th day after the mother’s discharge from hospital. In Yanchi, there is one village doctor in every village but no MCH worker in any of the villages, so the village doctors are required to do the work of both.

### Participants

To understand HCP’s perceived barriers to the provision of breastfeeding support in Datong and Yanchi counties, purposive sampling was used to recruit a total of 41 HCPs in the Tri-Level Healthcare System as research participants. Semi-structured IDIs were conducted with 21 participants, and 20 additional participants were recruited for five FGDs in Datong and Yanchi. Each focus group included 3 to 6 participants from the same health profession, except for one focus group conducted at the Yanchi Township level, which had a mix of township obstetricians and MCH workers. According to our observations, all participants contributed significantly to the focus group conversations.

Details of the participants are shown in Table 1.

**Table 1:** Participant details (*n* = 41)

Site	Level of health institution	Position of healthcare providers	No. of in-depth interviews	No. of focus group discussions	No. of focus group discussions participants	Total
Datong	County	Obstetrician-gynecologist	3	0	0	3
		Nurse	1	0	0	1
		Midwife	0	1	3	3
	Township	Obstetrician	1	0	0	1
	Village	Village doctor	4	0	0	4
		Village MCH worker	2	2	3/6	11
Yanchi	County	Obstetrician	4	0	0	4
		Nurse	1	0	0	1
	Township	Obstetrician	1	1(mix with obstetricians and township MCH workers)	2	3
		Township MCH worker	1		1	2
	Village	Village doctor	3	1	5	8
Total			21	5	20	41

### Data collection procedures

All participants were informed of the purpose of the study and told that their participation was voluntary and they could withdraw at any time. Once participants understood the study’s aim and were assured of confidentiality, consent was solicited by the researchers. With assistance from local health staff, the researchers recruited participants and interviewed them during the period from March 2018 to August 2018. All interviewers and researchers involved in this study were highly experienced, having undergone thorough training in interview techniques and data collection prior to the commencement of the research. We first organized the FGDs to obtain entry into the community and identify areas of consensus and disagreement concerning challenges of breastfeeding support. We then took the themes developed in FGDs to narrow in and probe more deeply through IDIs.

Each interview was conducted in Mandarin by an interviewer and a research assistant. The interviewer asked questions, and the assistant took notes. The in-depth semi-structured interviews included questions about the participants’ daily work routine, their experiences of providing breastfeeding support, the training they received about breastfeeding, barriers and facilitators they encountered when providing breastfeeding support, and suggestions for future breastfeeding health services. Due to time constraints, the specific questions asked varied between the two methods. FGDs questions were more concise while IDIs allowed us to dig deeper into the questions and explore more contextual information. All FGDs were conducted in the hospital conference rooms. All IDIs were conducted at locations guaranteeing privacy where participants felt comfortable, such as participants’ homes and hotel conference rooms.

Sampling and data collection continued until saturation was reached. The length of FGDs ranged from 52 to 108 min, and the IDIs ranged from 44 to 80 min. All the interviews were audio-recorded, transcribed verbatim and checked for accuracy. To protect confidentiality, audio files were deleted after transcriptions were completed; moreover, any identifying information from the interviews was deleted. Every participant received a small thank-you gift.

### Data analysis

The manuscripts were firstly transcribed in Mandarin and then entered into ATLAS.ti for coding and analysis. Data were analysed thematically. Three researchers (J. Wu, Q.Zhang and J.Huang) discussed and developed a code list after reading several transcriptions. Each of them independently coded three interviews and discussed discrepancies in applying codes until consensus and high inter-coder reliability were reached. Then all remaining transcriptions were coded by three researchers independently, with FGDs and IDIs being coded separately. Emerging subthemes, themes, and relationships among the codes within and across documents were sought. The authors collectively defined four final themes that were re-checked against the transcribed data and codes. Findings from FGDs and IDIs are consistent and thus are presented together. The translation of the quotation into English was carried out by the first author (J. Wu) and cross-checked by co-authors.

## Results

### Characteristics of the participants

The demographic information of the participants, including the highest degree obtained, gender and position holding, is summarized in Table 2.

**Table 2:** Participant demographic information (*n* = 41)

Variable			County	Township	Village
Age (Median(interquartile range))			43.5 (32.3,48.8)	40 (24.8,48.5)	45 (27,53)
Education	General education	Below high school	0	0	8
		High school	0	0	1
		College	0	1	0
	Professional training	Technical school	3	4	11
		Junior medical college	1	1	2
		Medical college	8	0	1
Gender		Female	12	5	15
		Male	0	1	8
Position		Obstetrician-gynecologist	7	4	0
		Midwife	3	0	0
		Nurse	2	0	0
		Village doctor	0	0	12
		Township MCH worker	0	2	0
		Village MCH worker	0	0	11
Years in position (Median (interquartile range))			13.5 (6.3,25.5)	14 (1.8,22)	10 (4,21.5)

### HCP’s perceived barriers to providing breastfeeding support

Our analyses revealed four themes that the participants perceived as barriers to supporting breastfeeding. In each of these themes, several subthemes were also identified. The themes and subthemes are summarised in Table 3 and described with representative quotes presented below.

**Table 3:** Themes and subthemes of HCP’s perceived barriers to breastfeeding support

Themes	Subthemes
(1) Lack of medical resources	Inadequate staffing Lack of financial incentives
(2) Lack of clear and specific responsibility assignment	No one takes the lead Mutual buck-passing
(3) HCP’s lack of relevant expertise	Lack of knowledge and skills Low prestige of village HCPs
(4) Difficulties in accessing mothers	Medical equipment shortages reduce services utilisation Mothers’ housing situation Mothers’ mobility Cultural barriers

### Lack of medical resources

#### Inadequate staffing

Although county-level hospitals were crucial places where almost all mothers went to give birth, these hospitals faced serious shortages of healthcare practitioners. This led to heavy workloads and left HCPs with no extra energy or time to support breastfeeding. Compared to supporting breastfeeding, which is perceived as relatively less urgent and is rarely a life-or-death matter, completing the most critical work and ensuring the safety of the mothers were HCPs priorities. As one participant reported:

*We don’t have enough doctors, midwives, and nurses. There are often three- or four-women giving birth simultaneously (in the delivery rooms), with only two midwives on duty. The midwives need to pay attention to priorities such as whether the mother is hemorrhaging or the newborn is suffocating. So, many other details such as delayed cord clamping, early skin contact, and early initiation of breastfeeding cannot be implemented. (obstetrician, county-level)*

Staff shortage result in insufficient time available for practitioners to support breastfeeding, and “lack of time” and “heavy workload” were the most common complaints by HCPs. As a county obstetrician reported:

*There are about 70 pregnant women registered to be seen in one morning. The time allocated to each patient is so limited that I have no extra time to provide breastfeeding support. I will pay attention to these details if I have spare time.*

In addition, medical training among township and village HCPs was extremely inadequate. Some HCPs had never received any formal education, others had obtained a junior high school or high school education and took up their jobs after completing a short-term medical training course provided by local health authorities. Those HCPs with less formal education and training described reduced ability to provide breastfeeding support. As one participant expressed:

*I haven’t finished primary school and can hardly read, it’s hard for me to provide professional health services for mothers. (village doctor)*

### *Lack of financial incentives*

According to participants working in county hospitals, lack of financial incentives also negatively impacted HCP’s provision of breastfeeding support. Breastfeeding support was provided free of charge to women and did not bring in any revenue for the hospitals. HCPs were expected to provide these time-consuming services without additional payment, which affected their enthusiasm and devotion to providing breastfeeding support. As one county nurse reported:

*It (breastfeeding support) takes a lot of time and does not make any profit, we do it for free!*

Further, village HCPs were generally not well-paid, and they were not motivated to dedicate themselves to health services. They also needed to make their living by doing other work. Low pay resulted in a high turnover rate for village doctors which further contributed to the shortage of village doctors and exacerbated the healthcare crisis for mothers and babies.

*Village doctors are farmers. They are paid very low wages and are not provided with any of the five social benefits mandated by the Chinese Government (including a pension, health insurance, unemployment insurance, occupational injury insurance, and housing funds). (obstetrician, township-level)*

*They also raise cattle or run a business to earn money to support their families. Some village doctors have quit their jobs because of the low wages for their medical services. There is a shortage of village doctors, and the quality of MCH service is still a big concern in the village. (obstetrician, township-level)*

### Lack of clear and specific responsibility assignment

#### No one takes the lead

The general absence of administrative support for prioritizing breastfeeding services results in ambiguity regarding the entity or individual responsibility for overseeing overall breastfeeding support. As one participant stated:

*The problem is that nobody takes the lead in providing breastfeeding support across the entire care continuum. We just do what we can do. (nurse, county-level)*

Hospital administrators paid more attention to improving hospital reputation and to increasing profits. Some participants expressed that administrators do not perceive breastfeeding support as important.

*Our leaders do not know what efforts need to be taken to promote breastfeeding, nor do they realise how hard it is. Actually, we have many challenges, but they do not care. Much detailed work (to promote breastfeeding) cannot be carried out. (nurse, county-level)*

### *Mutual buck-passing*

Due to the lack of clear and specific assignment of responsibility, all HCPs endorsed a general sense of breastfeeding support as a shared responsibility, but did not know what specific roles they should take. As a result, HCPs were reluctant to take the responsibility and this actually led to mutual buck-passing.

HCPs in county hospitals relied on each other to provide breastfeeding support. For example, county inpatient HCPs complained that mothers knew nothing about breastfeeding and were not ready for breastfeeding. They criticized county outpatient HCPs for not preparing pregnant women for breastfeeding readiness.

*Many women take prenatal checkups in the county hospital outpatient* service. *If someone gives them breastfeeding guidance, then it will be easier for us when the mothers are hospitalised for giving birth. (nurse, county-level)*

*For mothers with clogged milk ducts, if we have enough time, we may help them, otherwise we can’t. But this is also the responsibility of nurses. (obstetrician, county-level)*

Meanwhile, county outpatient HCPs expected township and village HCPs to provide more breastfeeding support prenatally.

*Women are always in the third trimester when they come here. Township and village HCPs have more time and opportunities to communicate with mothers. They should do a good job of breastfeeding support. (obstetrician, county-level)*

At the same time, township and village HCPs believed that insufficient knowledge and skills prevented them from supporting breastfeeding. They often recommended that mothers go to upper-level hospitals for treatment or assistance when encountering breastfeeding problems.

*There was a mother who had breast pain and could not feed the baby. I did not know how to help her, so I told her to go to the county hospital for treatment. (village doctor)*

### HCP’s lack of relevant expertise

#### Lack of knowledge and skills

Participants described lack of knowledge and skills as a major barrier to breastfeeding support. HCPs, especially those working in townships and villages, often did not have basic breastfeeding knowledge. One village MCH worker shared her misconceptions about breastfeeding:

*Anyway, I do not approve of breastfeeding when mothers got mastitis because the quality of breast milk is not good for baby feeding.*

Participants attributed their overall lack of breastfeeding knowledge and skills to inadequate training.

*We never participated in breastfeeding-related training. Breastfeeding knowledge and skills are rarely involved in our re-educational trainings. (obstetrician, county-level)*

HCPs who did receive breastfeeding training, attributed its lack of effectiveness to improper training methods.

*I received training before. The trainers taught too much and too fast. Now, I can hardly remember what I have learned, and I never used those in my practice. (village MCH worker)*

### *Low prestige of village HCPs*

As stated earlier, village HCPs were usually not well trained in modern medicine and had insufficient breastfeeding knowledge and skills. Moreover, they were not familiar with the new mothers, which eroded their authority and further hindered them from providing breastfeeding support. Some village HCPs reported that mothers did not trust what they said. One village MCH worker described her frustrations this way:

*I don’t know much about breastfeeding. When I saw relevant information on the internet, I forwarded it to the Wechat group chat. But I guess they didn’t pay attention to these messages at all. No one ever asked me any question.*

### Difficulties in accessing mothers

#### Medical equipment shortages reduce services utilisation

Both physicians and mothers increasingly value detailed prenatal examinations like four-dimensional color Doppler ultrasounds. However, such medical equipment is not always available in every healthcare facilitates. Medical equipment shortages are especially common in township or village clinics. These lower-level HCPs are in close geographic proximity to women and have the potential to be the first stop for women in accessing breastfeeding support services. However, the medical equipment shortages in township and village care systems compels mothers to travel to more distant locations seeking prenatal health services. In addition, mothers often have to visit multiple HCPs to complete prenatal checkups. Consequently, there are missed opportunities for HCPs in lower-level healthcare systems to establish and maintain contact with mothers to provide continuous care.

*We can only cope with basic examinations such as blood pressure, fetal heart rate, and B-ultrasound, so we usually tell them to go to the county hospitals to do other tests. Mothers also need to go to the provincial hospitals to do four-dimensional color Doppler ultrasounds and Down’s syndrome screening because the county hospitals don’t have the equipment. (obstetrician, township-level)*

### *Mothers’ housing situation*

Mountainous, remote, and dispersed living arrangements made it difficult for mothers to access medical services. Mothers were described as *“reluctant or unable to travel the long distance to get help for an urgent breastfeeding problem”.* Participants also described how they performed postpartum visits within the constraints of the geographical location.

*The villagers’ residences are very remote and dispersed. Some even live in the mountains. I need to ride 15 km to get to the farthest family. When it rains, I have to walk for half the distance. (village doctor)*

In addition, in rural areas, many families kept watchdogs for security, which created safety threats for HCPs when conducting postpartum visits.

*If there is a watchdog waiting for me at the gate, that’s big trouble. The first thing to do after getting off the motorcycle is to find something to fight with the dog. (village doctor)*

*The most difficult thing for me is the watchdog. I usually spend one or half an hour getting to the mother’s home but dare not get in because of the watchdog. (village MCH worker)*

### *Mothers’ mobility*

Due to severe unemployment in rural areas, many Chinese work and live in big cities far from their hometowns, including pregnant women. Pregnant women, however, do return to their hometowns to give birth because they can be taken care of by their own mothers and relatives, and also because the cost of giving birth at the local county-level hospital is highly reimbursable by the government. However, some do not return until the third trimester and complete their prenatal checkups elsewhere. Some of them will even return to the city for work two or three months after giving birth, at which point they will have stopped breastfeeding. Thus, mothers’ mobility due to working in distant cities is a barrier to HCPs ability to understand mothers’ physical conditions and to build relationships to provide continuous anticipatory guidance for mothers.

*Mothers have to work in big cities even if they are pregnant. When they come back, they are about to give birth. Their prenatal checkups are not done here. That is troublesome for us (obstetrician, township-level).*

*Most mothers are not at home. I seldomly know them and have never met some of them. We communicate through the cell phone (village doctor).*

### *Cultural barriers*

Mothers’ resistance to breastfeeding due to cultural beliefs and practices that contradict breastfeeding recommendations also influenced HCP’s breastfeeding support. *Zuoyuezi* is a traditional norm requiring women to stay in bed or at least indoors for the first month after giving birth. During this time, non-family members are usually not allowed to visit the mother and baby, making it difficult for HCPs to conduct postpartum home visits. Some village HCPs are males, who are particularly unacceptable to handle problems of breastfeeding. The problem is more severe when there are no other female HCPs in villages as was the case in Yanchi county.

*When I went for postpartum visits, the mothers and babies always stayed in a room, and they would not let me in for fear that their babies would cry (village MCH worker).*

Mothers belonging to ethnic minorities like the Hui would not follow checkups due to their cultural beliefs, making it difficult for HCPs to know what was happening to these mothers.

*They do not like others to look at their bodies. They said that their body would be dirty after taking prenatal checkups (obstetrician, township-level).*

## Discussion

Despite the goal of China’s Tri-Level Healthcare System to provide breastfeeding support from prenatal to postpartum stages, this study found that in two northwest rural counties, HCPs faced significant barriers to providing this support at the local level. Similar to findings from studies conducted in other countries, this study also found lack of medical resources to be a crucial barrier, although difficult to surmount [13, 15].

Our study also found that the lack of clear and specific assigned responsibility is a major barrier, which may be caused by ambiguity in each given Chinese institutions and HCP’s breastfeeding support responsibility. In western countries such as USA and Australia, the specific breastfeeding-support responsibilities of different HCPs are defined. The American Academy of Pediatrics (AAP) and the American College of Obstetrics and Gynecology (ACOG) call for their members, including paediatricians, obstetrician-gynecologists, IBCLCs, and paediatric nurse practitioners, to be at the forefront of providing breastfeeding support [21–23]. In Australia, midwives and MCH nurses in the community have a clear division of responsibilities according to stage of pregnancy. In addition, mothers can seek extra breastfeeding support from lactation consultants at specialist infant feeding clinics or the Australian Breastfeeding Association [24].

Furthermore, HCPs in our study described how they were constrained by difficulties in accessing mothers, a problem also reported in other studies [14]. Studies have shown that mobile health (mHealth)-based interventions such as web-page education or smartphone applications may help offset some of the difficulties associated with access. MHealth interventions can provide much needed education about breastfeeding to all women, specifically to low-income, rural women, and thus contribute to improvements in breastfeeding rates [25, 26]. The advantages of mHealth-based interventions include portability, timeliness, efficiency, and scalability.

### Recommendation

Our study highlights the adverse effects of insufficient medical resources on HCP’s breastfeeding support in rural areas of China. This underscores the need to reevaluate the importance of providing adequate medical staff and appropriate rewards for breastfeeding support, including financial incentives and a sense of achievement. The limitations of social and economic development, low wage compensation for village HCPs, and inadequate staffing, however, may not be overcome in a short period of time [27]. Nonetheless, if financial and other incentives such as career development opportunities, reward and recognition programs were in place, China can also refer to the professional guidelines released by ACOG and AAP for clarification of roles and responsibilities useful in ensuring that proper support to mothers is provided at multiple levels.

However, other barriers may be surmountable at present to promote breastfeeding. In recent years, the Chinese government has been actively promoting mobile medicine. Chinese researcher Liu et al. reported the practices of a smartphone application-based clinic in Guangzhou Women and Children’s Medical Centre. The nurses offered 24-hour live services for women and children through text, graphics, voice, video, and video chat, or provided drop-in services according to the patient’s need. The study found that 99.52% of the mothers were satisfied with the services [28]. Cell phones were already widely used among mothers and HCPs in our study. mHealth-based interventions may be promising approaches for HCPs to deliver breastfeeding support to mothers in rural China. However, what specific health personnel should provide support, what their responsibilities should be, what would be effective content and what approaches the intervention should take, all still require further study.

In sum, mHealth-based interventions are available and can be further developed, refined and expanded as an immediate and cost-effective solution. Long-term solutions, including improving financial benefits and other incentives for breastfeeding support and clarifying roles and responsibilities, still necessitate additional resources.

### Limitations of the study

We recognize some limitations of the study. Due to recruitment difficulties, we have limited participants from leadership, such as directors of obstetrics departments and hospital directors. Therefore, the breastfeeding support barriers they perceive in the Tri-Level Healthcare System were not adequately represented in our data. Due to the lack of these people’s perspectives, suggestions given in the discussion section may need further investigation. Additionally, the study only investigated HCPs in two counties in northwest China, which have distinct geographic and demographic features outlined in the setting section. Though many rural communities in other regions in China, especially in Northwestern areas, share similar institutional context (e.g. Tri-level Healthcare System management and operation style, staffing challenges etc.) and community context (e.g. Mothers’ mobility, sparsely populated rural condition etc.), because Northwest China is a vast region with diverse demographic features, other contextual factors may exist and influence HCP perceived barriers, which is not found in our study.

## Conclusion

In this study, HCP’s perceived barriers to providing breastfeeding support in rural Northwestern China were identified, including lack of medical resources, lack of clear and specific responsibility assignment, HCP’s lack of relevant expertise and difficulties in accessing mothers. Considering the unique structure of the Tri-Level Healthcare System, some perceived barriers, particularly inadequate staffing, and lack of financial incentives, are not surmountable in a short period of time. We offered some feasible suggestions that may promote breastfeeding in the near and long term. We recommend that researchers should explore improvement in mHealth-based intervention efficiency such as content and platform. Also, when financial and other incentives are in place, breastfeeding support roles of HCPs who serve women and children in different settings should be clarified by the Tri-Level Healthcare System. Along with more clearly defined roles, proper training is needed for HCPs to carry out their specific responsibilities. Further research is needed to understand and evaluate the viability of these approaches in rural Northwestern China and other rural underdeveloped and remote areas within the country.

## Data Availability

The datasets used and/or analysed during the current study are available from the corresponding author on reasonable request.
